# Multiplexed High-Throughput Serological Assay for Human Enteroviruses

**DOI:** 10.3390/microorganisms8060963

**Published:** 2020-06-26

**Authors:** Niila V. V. Saarinen, Jussi Lehtonen, Riitta Veijola, Johanna Lempainen, Mikael Knip, Heikki Hyöty, Olli H. Laitinen, Vesa P. Hytönen

**Affiliations:** 1 Faculty of Medicine and Health Technology, Tampere University, 33520 Tampere, Finland; niila.saarinen@tuni.fi (N.V.V.S.); jussi.lehtonen@tuni.fi (J.L.); heikki.hyoty@tuni.fi (H.H.); olli.laitinen@tuni.fi (O.H.L.); 2 Department of Paediatrics, University of Oulu, 90570 Oulu, Finland; riitta.veijola@oulu.fi; 3 Department of Paediatrics, University of Turku, 20520 Turku, Finland; johanna.lempainen@utu.fi; 4 Pediatric Research Center, Children’s Hospital, University of Helsinki and Helsinki University Hospital, 00029 Helsinki, Finland; mikael.knip@helsinki.fi; 5 Research Program for Clinical and Molecular Metabolism, Faculty of Medicine, University of Helsinki, 00029 Helsinki, Finland; 6 Fimlab Laboratories, 33520 Tampere, Finland

**Keywords:** enterovirus, rhinovirus, multiplex immunoassay

## Abstract

Immunological assays detecting antibodies against enteroviruses typically use a single enterovirus serotype as antigen. This limits the ability of such assays to detect antibodies against different enterovirus types and to detect possible type-specific variation in antibody responses. We set out to develop a multiplexed assay for simultaneous detection of antibodies against multiple enterovirus and rhinovirus types encompassing all human infecting species. Seven recombinant VP1 proteins from enteroviruses EV-A to EV-D and rhinoviruses RV-A to RV-C species were produced. Using Meso Scale Diagnostics U-PLEX platform we were able to study antibody reactions against these proteins as well as non-structural enterovirus proteins in a single well with 140 human serum samples. Adults had on average 33-fold stronger antibody responses to these antigens (*p* < 10^−11^) compared to children, but children had less cross-reactivity between different enterovirus types. The results suggest that this new high-throughput assay offers clear benefits in the evaluation of humoral enterovirus immunity in children, giving more exact information than assays that are based on a single enterovirus type as antigen.

## 1. Introduction

Enteroviruses (EV) constitute a large group of common non-enveloped RNA viruses that cause a wide variety of diseases ranging from mild skin conditions and respiratory problems to more severe inflammation in the heart [1], central nervous system [2], or the pancreas [3]. Infections in young infants can be life-threatening and infections at any age can cause irreparable damage. The hand, foot, and mouth disease epidemics are also frequently caused by enteroviruses and have led to severe encephalitis especially in China and Southeast Asia [4]. While the infections caused by enteroviruses are common and there are more than 200 known serotypes of enteroviruses, there are not many serological tools with wide coverage for diagnosing and studying them. Currently, reverse transcription polymerase chain reaction (RT-PCR) is widely used to diagnose these infections by detecting viral RNA in clinical samples. RT-PCR is a valuable tool when the sample can be taken at the acute phase of infection, but it cannot detect already resolved recent infections and it does not routinely allow identification of the serotype that caused the infection [5,6]. This can lead to wrong/false negative findings as the virus is present in a clinical sample only for a few days [7]. Species- or strain-specific identification of enteroviruses is mainly performed by sequencing, which is both labor intensive and costly.

In acute infections, such as enteroviral myocarditis, that causes mortality especially in neonates, a fast diagnosis is important to immediately start the appropriate treatment [8]. Viral myocarditis is hard to distinguish from other causes of myocarditis, and reliable diagnosis is important to offer the patients right treatment [9]. However, direct detection of the virus is often difficult due the delay from acute infection to the onset of symptoms of myocarditis, and serological assays are frequently used to screen enterovirus infections from these patients.

Enterovirus infections have also been linked to chronic diseases. For example, they have been linked to the development of chronic dilated cardiomyopathy, [10,11] type 1 diabetes [12,13,14,15], and chronic pancreatitis [3], which can also be a risk factor for pancreatic cancer [16]. Rhinovirus (RV) infections have been linked to asthma exacerbations [17]. Studies on possible role of enterovirus infections in chronic diseases require large prospective follow-up cohort follow-ups where infections should be diagnosed long before the disease onset. In such studies, serological assays offer clear benefits since the samples do not need to be taken during the acute phase of the infection. This is particularly important aspect in enterovirus infections since the majority of acute infections are subclinical.

Enterovirus species that infect humans include enteroviruses A through D (EV-A, EV-B, EV-C, EV-D) and rhinoviruses A through C (RV-A, RV-B, RV-C). Since enterovirus VP1 capsid proteins have the most sequence variation between serotypes and species, they are theoretically the best option as antigen in assays aiming at detection of antibody responses against different enterovirus types. However, they also contain the enterovirus group-specific *N*-terminal antigenic epitope, which binds antibodies that have been induced by a wide variety of different enteroviruses [18]. We set out to see if a panel of VP1-proteins from representatives of all human infecting enterovirus species could be used to make an assay that can identify antibody responses to enteroviruses on a species level. To this panel of species-specific antigens, we added conserved non-structural enterovirus proteins that we have shown in a previous study to act as markers for an acute enterovirus infection in adults [19].

Prototype viruses representing different species of human enterovirus and rhinovirus including CVA4 (EV-A), CVB1 (EV-B), PV1 (EV-C), EV-D68 (EV-D), RV-A89, RV-B14, and RV-C3 (nomenclature according to International Committee on Taxonomy of Viruses (ICTV)) were selected based on their previous use as vaccines or due to their pathogenicity. For example, PV1 was chosen since vaccinating for it is a part of the national vaccination program in many countries and EV-D68 was selected due to the serious epidemics in Western countries in recent years [20]. RV-A89 and RV-B14 were picked since they are used in prototype vaccines and have been shown to produce cross-neutralizing antibodies [21] and RV-C3 was chosen because this serotype has been associated with asthma [22,23].

**Figure 1:**
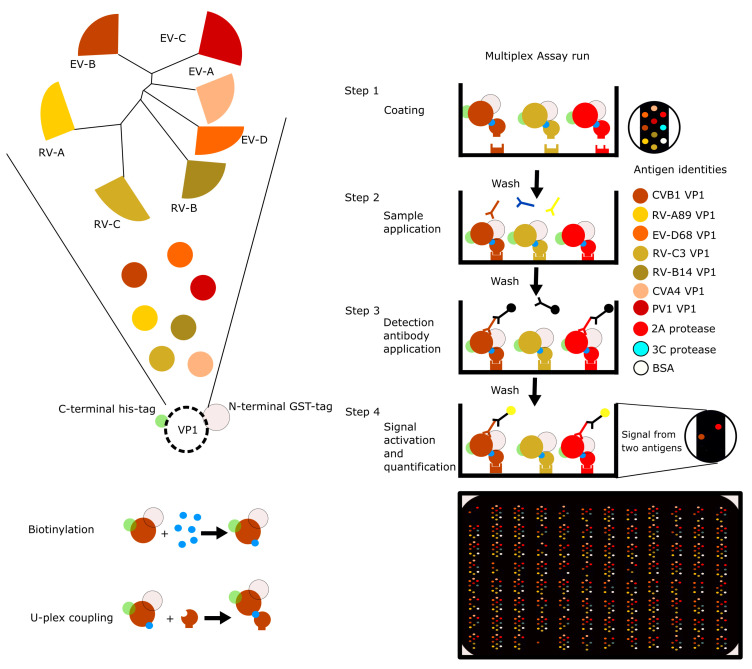
Multiplex assay development. Representative VP1 protein sequences from all human infecting enterovirus and rhinovirus species were equipped with an N-terminal GST-tag and a C-terminal 6xhis-tag for purification after bacterial expression. The purified recombinant VP1 proteins along with 2A and 3C proteases from CVB3 and a BSA control were biotinylated and each antigen was coupled to a unique U-PLEX linker. During coating each U-PLEX linker coupled antigen would target a specific site at the bottom of the well. After coating, the assay is run like a regular indirect ELISA, except for having a secondary antibody with electrochemiluminescence tag, and a suitable substrate and an MSD plate reader.

As testing for multiple antigens using traditional ELISA, especially with an extensive set of samples, is very labor intensive, and multiplexing platforms based on spotting cannot be changed once the antigens have been plated, we decided to use something more flexible, and adapted the test to Meso Scale Diagnostics (MSD) U-PLEX linker-based platform (Figure 1) which is simple, fast, and allowed us to change the antigens on the fly. The resulting multiplexed serological assay is suitable for evaluating the serological history with low sample volumes and can be quickly adapted to suit different needs by changing individual antigens.

## 2. Materials and Methods

### 2.1. Production and Purification of Enterovirus Antigens

The proteases 2A and 3C were produced as described in [24]. Briefly, 2A and 3C sequences were ordered as artificial genes from Life Technologies and cloned to pBVboostFGII [25] expression vector. The proteins were expressed in BL21-AI (Life Technologies, Espoo, Finland) *Escherichia coli* (*E. coli*) strain and purified using HisTrap FF crude immobilized metal affinity chromatography columns (GE Healthcare Bio-Science AB, Umeå, Sweden) [24].

VP1 sequences from EV-A, EV-B, EV-C, and EV-D (CVA-4, CVB1, PV1, EV-D68) and RV-A, RV-B, and RV-C (RV-A89, RV-B14, RV-C3) were obtained from online databases (Table A1).

The sequences were obtained as synthetic gene strings from GeneArt. Hexahistidine-tags were added to the 5′ end of the VP1s and the resulting sequences were flanked with restriction sites (BamHI and EcoRI), as well as generic sequences for PCR-amplification (not included into expression cassette). The gene strings were amplified with PCR and purified from agarose gel-electrophoresis. Both the purified VP1 sequences and pGEX-2T vectors were cut with FastDigest Restriction Enzymes BamHI and EcoRI (Thermo Scientific, Loughborough, UK), the plasmid was phosphatase treated with FastAP (Thermo Scientific, Loughborough, UK), and the cut fragments were purified using agarose gel electrophoresis. Ligation reactions were transformed into chemically competent Top 10 *E. coli* cells using heat shock method and plated onto LB-Amp plates. Colonies were picked, minipreps prepared and DNA-sequenced with in house Sanger sequencing service. The amino acid sequences of all the recombinant VP1 proteins have been listed for easy comparison in Table A2.

For protein production, the verified plasmids were transformed into BL21 Star (DE3) *E. coli* and small seed cultures prepared overnight at +37 °C. Larger expression cultures were inoculated with the seed cultures and grown to OD600 0.6–0.8 at 37 °C and the VP1 production induced using 100 µM IPTG. The culturing temperature was then lowered to 28 °C and the cultures were harvested the following day. The bacteria were pelleted by 20 min centrifugation at 4,000 rcf. The pellet was resuspended into pH 8.0 PBS and the bacteria lysed using sonication. The lysate was clarified by 20 min centrifugation at 25,000 rcf and the supernatant incubated overnight with glutathione resin at +4 °C on shaking. The resin was washed with the binding buffer and the protein eluted with a Tris buffer (50 mM Tris-HCl 500 mM NaCl, pH 8) including 40 mM glutathione and 20 mM imidazole. VP1 containing fractions were pooled and bound to Histrap resin for 1 h at RT, washed with Tris buffer with 30% glycerol and eluted with Tris buffer containing 500 mM imidazole.

Samples from each purification step were run on SDS-PAGE Criterion TGX stain-free gels and transferred to nitrocellulose for Western blotting to analyze the purification process and the purities of the final products. Nanogam a pooled human serum concentrate and a HRP-conjugated rabbit anti-human IgG secondary antibody (Dako cytomations P0214) were used for detection. Protein concentrations were measured using UV-VIS spectroscopy and Pierce BCA protein assay kit (23225).

Proteins were dialyzed into PBS pH 7.4 and biotinylated for 3 h with 20× molar excess of EZ-link biotin reagent (Thermo Scientific, Rockford, IL, U.S.A., catalogue number #35389) at RT. Unbound biotin was removed by dialysis. Relative degree of biotinylation was analyzed densitometrically with a procedure resembling Western blotting: 10 µg of purified, biotinylated antigen were loaded on ready–made BioRad Criterion TGX stain-free gels. Monobiotinylated BSA (biotinylation quantified with a B4F assay [26]) was loaded (10 µg, 20 µg, 40 µg, 80 µg) onto the gel as a standard. After transfer, the BSA-blocked membranes were incubated with 10 nM neutral chimeric avidin [27] o/n at +4 °C. After a washing step an in-house rabbit-anti-avidin polyclonal antibody was used to detect the neutral chimeric avidin bound to the biotnylated antigens. Unbound antibodies were washed away and an HRP-conjugated horse-anti-rabbit antibody was used as a detection antibody. ECL reagent was added to the washed membrane and the resulting chemiluminescence was imaged and densitometrically quantified using Biorad Chemidoc instrument with Image Lab software. Nanogam was used as a positive control sample to detect VP1 antigens in Western blot.

### 2.2. Setting Up the Multiplex Assay

U-PLEX Development Pack was obtained from Meso Scale Discovery. Linker reactions were modified to the U-PLEX 10 kit standard so that molar excess of linkers to antigens was adjusted based on the amount of biotins/antigen. For example: If antigen had four biotins, we used the recommended amount of protein and if the antigen had three biotins, 1.33 times the recommended amount of the biotinylated protein was included.

Linking reactions were incubated for 1 h at RT after which they were quenched using the stopping buffer provided in the kit and equal volumes of each linker reaction solution were pooled together to make the coating solution.

Fifty microliters of the coating solution was added to each well and incubated for 1 h RT with shaking. The wells were washed thrice with 150 µL PBST and blocked with 50 µL 1% BSA in PBS. After another washing step, the analytes were loaded in the wells.

To test the coating of the antigens, we incubated 10 nM sulfo-tagged neutral chimeric avidin in the wells for 1 h at RT on shaking, washed the wells, added 150 µL of 2× sulfo-tag substrate and ran the measurement with the MSD instrument. After receiving similar signals from all the antigens with the coating control, we tested our positive control Nanogam. Once we established that the pooled serum concentrate gave signal for all antigens, we moved on to analyzing the individual serum samples.

### 2.3. Human Serum Samples

Serum samples from patients with confirmed acute enterovirus infection (*n* = 20) were obtained from Turku University Hospital [19]. Control samples (*n* = 22) were collected from healthy individuals [19]. Another serum sample collection was obtained from the Diabetes prediction and prevention (DIPP) consortium [28], total of 88 serum samples, with partially known enterovirus histories [15]. Finally, we obtained 20 serum samples from healthy adults from Tampere biobank, sampled from individuals during routine checkups. Sample information has been summarized in Table 1.

### 2.4. Human Samples Ethics Statement

Enterovirus IgM positive serum samples were originally tested in the virus diagnostic laboratory in Turku University Hospital for the diagnosis of patients’ acute enterovirus infections. The samples were anonymized and used in the development of antibody tests. Control serum samples included laboratory personnel and the participants of the Autoimmune defense and living environment-study (ADELE) [29]. Serum samples from children were obtained from under two-year-old participants, who were participating in the Diabetes prediction and prevention-study (DIPP) [28]. All participants or their legal guardians gave informed consent.

### 2.5. Workflow for Antigen Preparation and Assay Run

After the production of each antigen and confirming their functionality individually in traditional ELISA following the same protocol used previously [19], we biotinylated the antigens, added unique U-PLEX linkers to them, pooled them prior to coating and followed the protocol outlined in Figure 1. After confirming with a limited sample set, including Nanogam, that we obtained reasonable signals for each of the antigens, we ran a more comprehensive set of samples.

### 2.6. Analysis of Results

Data analysis was carried out using R v. 3.6.1 in RStudio 1.2.1335 with the following packages: GGAlly, ggplot2, dplyr and reshape2. Wilcoxon rank-sum test was performed for comparing groups.

Cutoffs were calculated as in Equation (1)

Mean(x) + 3 × sd(x)(1)

where x is signal from dilution buffer BSA spots (four replicate wells).

Min-max normalization was calculated with the following formula (Equation (2)).

scaled(x) = (((b − a) × (x − min(x))) / (max(x) − min(x))) + a(2)

where x, is a signal from the distribution of signals for an antigen across all (adult or child) participants, a = 0 = desired minimum value after scaling and b = desired maximum value after scaling.

## 3. Results

### 3.1. VP1 Antigens Belonging to All Human Enterovirus and Rhinovirus Species Were Produced in E. coli and Characterized

**Figure 2:**
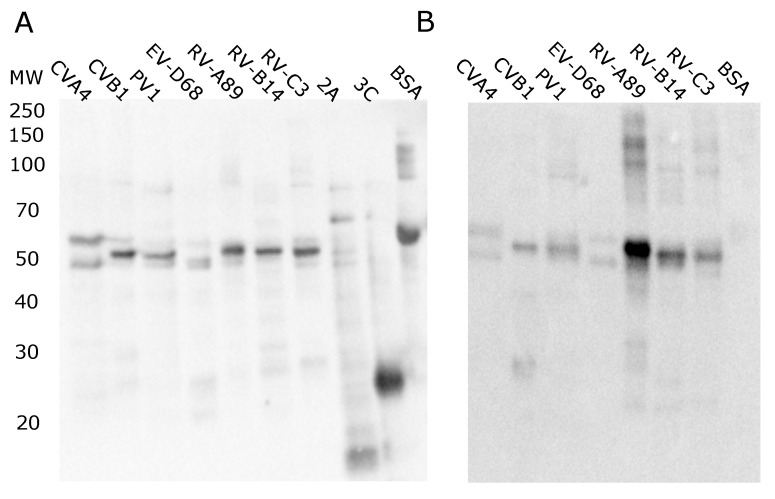
Antigen quality control. (**A**) The detection of biotinylation using neutral chimeric avidin and anti-avidin antibody. (**B**) Antigen detection in Western blot using pooled human serum (Nanogam).

**Figure A1:**
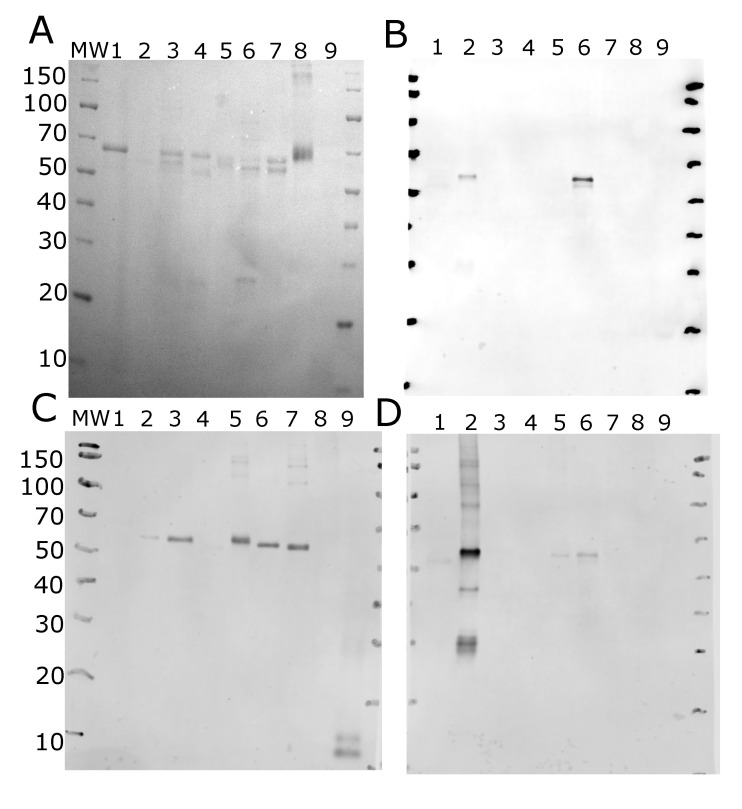
Western blot analysis for antigens. Lanes 1-7 are VP1 proteins for CVA4, CVB1, PV1 EV-D68, RV-A89, RV-B14, RV-C3, lane 8 is BSA, and lane 9 is 2A. Panel (**A)** shows the Ponceau-staining of the nitrocellulose membrane after transfer, (**B**) 3A6 anti-CVB1 monoclonal antibody, (**C**) anti-his antibody and (**D**) Dako 5D8/1.

VP1 proteins from all human infecting enterovirus species (EV-A to EV-D) as well as rhinovirus species (RV-A to RV-C) were expressed in *E. coli* and purified as described in materials and methods section, and their purity, biotinylation status (Figure 2A), and antigenicity (Figure 2B) were analyzed with Western blotting before they were used in the multiplex assay. For CVA4, CVB1 and EV-D68 VP1s we observed double bands, which we have seen previously for various VP1 proteins [30], and presumably the double band is a result of partial C-terminal proteolysis, as evident from Figure A1C, where anti-histag detected all VP1s. Also, Nanogam (which we have also used as positive control in the multiplex assay) recognized all VP1 proteins (Figure 2B). Nanogam detected the RV VP1s most strongly as expected from a pooled human serum concentrate, since rhinoviruses are extremely common. The bands observed between 20 and 30 kDa are most likely a result of proteolysis occurring between GST and the VP1 part of the fusion protein, as the size matches with VP1 and we observed 3A6 and Dako 5D8/1 monoclonal antibodies (both of which target the EV group specific epitope [18,30]) recognizing this band in CVB1 sample (Figure A1B,D). These analyses confirmed that the antigen preparations did not contain large amounts of impurities and that they are properly biotinylated to function as antigens in the U-PLEX assay.

### 3.2. Antibody Responses to VP1 Antigens Are Stronger in Acutely Infected Adults Than in Healthy Controls

**Figure 3:**
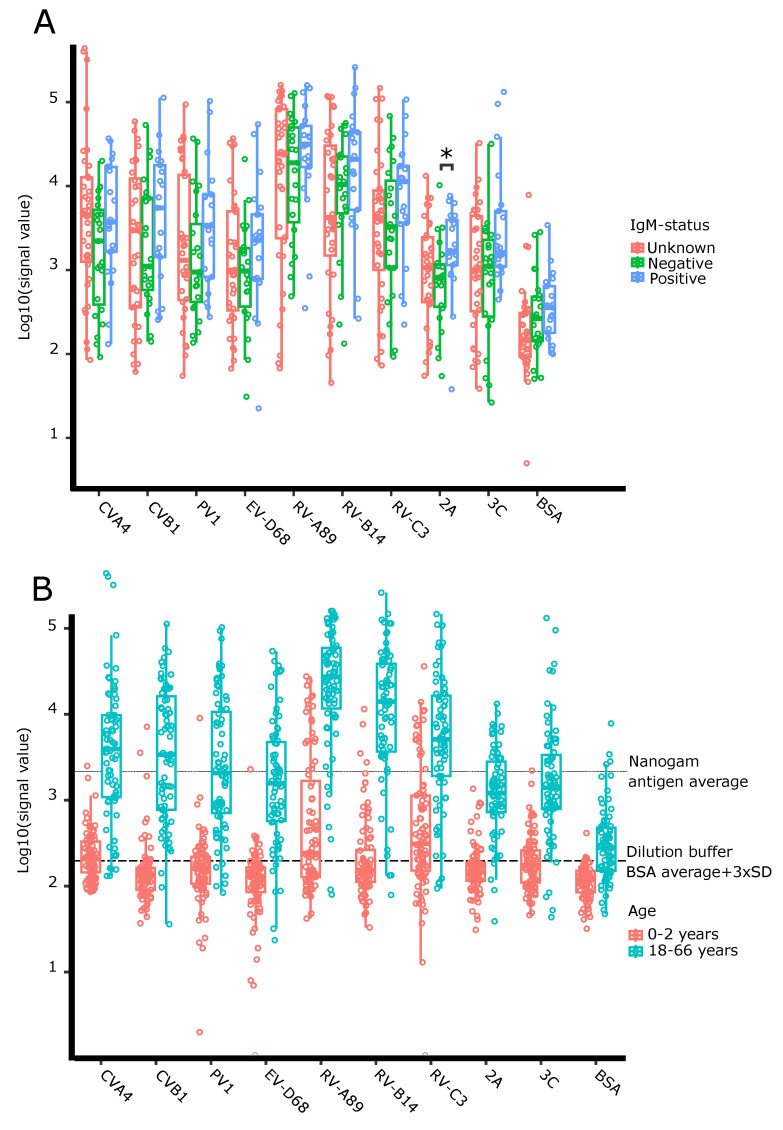
Different sample groups show variable raw signal ranges in antibody responses for EV and RV antigens in the multiplex assay. (**A**) Adult serum antibody responses grouped by IgM status. (**B**) Antibody responses grouped by the age groups of samples. * *p* < 0.05 in Wilcoxon rank-sum test after correcting for multiple testing (see Table 2).

We compared the antibody responses between the different adult groups based on their previous capture IgM assay results [19] (Figure 3A) as well as between adults and children (Figure 3B). Non-characterized adult serum samples collected from healthy individuals from Tampere biobank collection were included in the analysis as representatives of population background. The antibody responses towards EV and RV antigens are on average higher in IgM positive adult participants than in IgM negative controls (Figure 3A). Similarly, we observed the range of antibody responses in the previously non-characterized biobank samples overlapping both the responses in enterovirus IgM negative and IgM positive samples (Figure 3A). We conducted Wilcoxon rank-sum test to study the differences of IgM negative and positive groups and the found significant difference (*p* = 0.032) for the 2A protease response after Bonferroni correction (Table 2). Adults have on average 33 times higher VP1 and protease responses (*p* < 3.9×10^−11^, with Wilcoxon rank-sum test) than children under two years old (Figure 3B).

### 3.3. Antibody Responses to VP1 Antigens Are More Specific in Children Than in Adults

**Figure 4:**
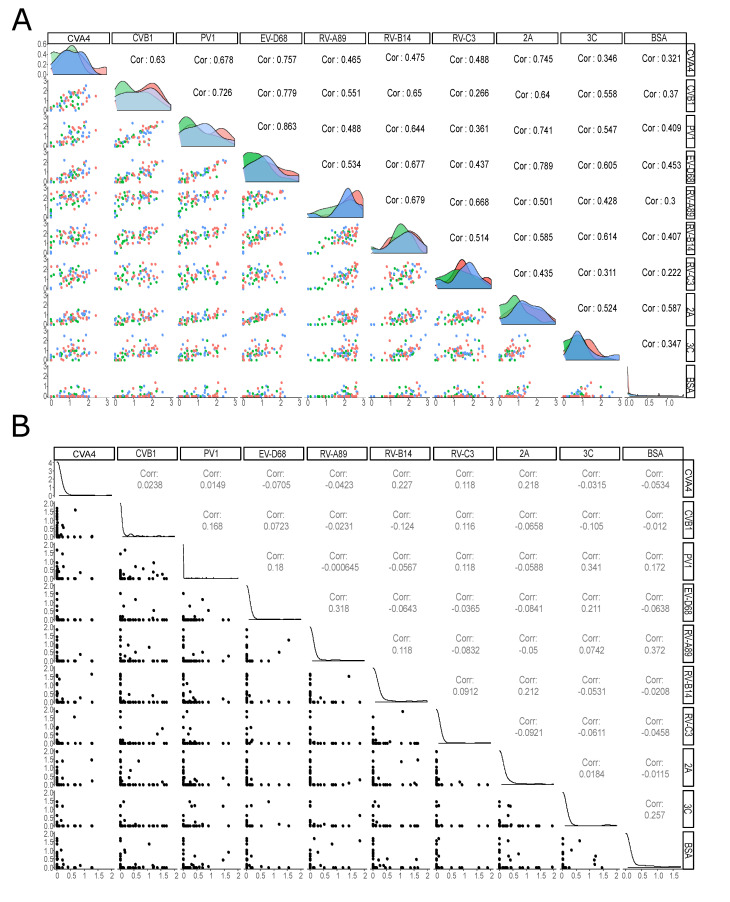
Scatterplots and correlations of antibody responses between different enterovirus antigens. The lower diagonal has scatterplots showing how pairs of antibody responses are correlated in (**A**) adults and (**B**) children and in the upper diagonal shows the corresponding Spearman correlation coefficients. In panel A, the diagonal shows the distribution of antibody responses grouped by IgM status: blue represents IgM positive group, green the IgM negative group and red the Tampere biobank samples collected from healthy adults, for which we do not know the IgM status, and which are representing the population background.

To study the specificity of the antibody reaction in the multiplex assay, we plotted the antibody responses to antigen pairs both in adults and children (Figure 4). To reduce the background noise, we considered everything below a log10(2.5) threshold (based on measured BSA signals from dilution buffer) to have a signal level of zero and subtracted the same value from remaining positive signals. Scatterplots in Figure 4A,B show that there is a great deal of cross-reactivity in the adult antibody responses, but very little in children. This difference is well summarized in the correlation coefficients for antigen pairs.

**Figure 5:**
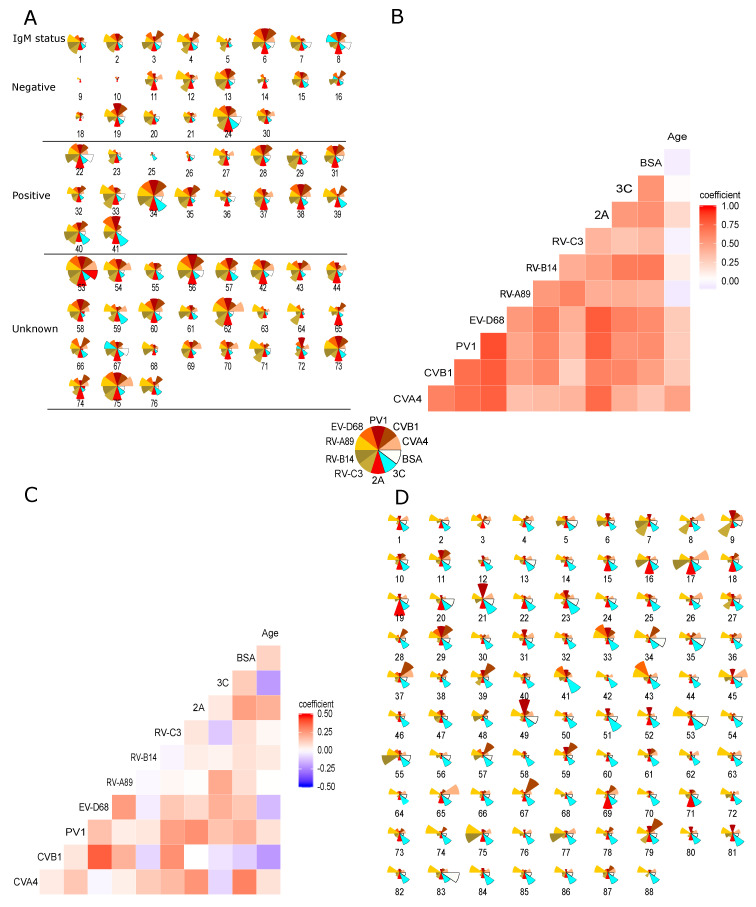
Antigen specific min-max normalized antibody responses and correlations of antibody responses between different enterovirus antigens. Antigen specific min-max normalized antibody responses (see Equation (2)) in the multiplex assay illustrated as radar plots for individual (**A**) adults and (**D**) children. Radii of the sectors represent normalized signal intensities. Age grouped Spearman correlations for antibody responses are illustrated as matrices for adults (**B**) and children (**D**). Min-max normalization was performed separately for adults and children, due to differences in signal levels in the two groups. Comparing (**A**) to (**D**) and (**B**) to (**C**), the differences between antibody responses in adults and children become apparent.

To investigate antibody responses at the level of individual participants, we plotted the min–max-normalized and scaled (Equation (2)) responses as radarplots for adults and children (Figure 5A,D respectively). To keep in mind the cross-reactivity of antibody responses, we also plotted the spearman correlation coefficients (Figure 5B,C). Figure 5A shows that the 20 IgM positive adults have on average higher 2A and/or 3C responses than the IgM negative ones, but it is hard to pick out the possible infecting agent based solely on VP1 responses as the signal levels are often similar for several ones simultaneously, regardless of IgM antibody status. This is also reflected in the correlation coefficients (Figure 5B), most notably for enteroviruses A–D. Comparing Figure 5B,C, which depict the correlation of antigen responses in adults and children, the antibody responses in children are less correlated than in adults. This is also evident when comparing individual responses (Figure 5A,D): In adults we typically see multiple high signals simultaneously, whereas in children there are only one or two high VP1 responses. Based on these results it seems like the antibody responses in children are more species specific than in adults, likely reflecting their more limited infection history.

## 4. Discussion

Immunological assays for studying enterovirus and rhinovirus infections when compared to PCR-based methods answer a different question. While RT-PCR can identify the presence of a virus, it will not tell anything about the immune response to it. Also, enteroviruses are detectable only for a short window of time in the patient’s blood with RT-PCR, whereas antibody responses can be much longer lived. On one hand RT-PCR requires larger sample volumes, which can be a problem, when running analysis from unique analytes with limited volume. On the other hand, due to the variability of antibody responses, setting a clear cutoff threshold for determining acute infections for viruses as common as enteroviruses and rhinoviruses can prove difficult and would require testing multiple samples with known infection histories confirmed by other means. We believe that with further testing we could use the antibody responses for non-structural proteins to assess how acute an infection is [19] and narrow down the culprit with the responses against VP1 proteins.

The U-PLEX multiplex assay based on VP1 antigens from human infecting enteroviruses and rhinoviruses produced interesting results. We showed that the antibody reactions to VP1 antigens were stronger and had more cross-reactivity in adults than in children. Because the antibody responses are more specific in children, this assay could be applied for testing cohort serum libraries collected primarily from children. Running this multiplex assay for samples that have already been characterized, initially to show repeatability of results, could further validate the specificity of the antibody responses. Such validation would also tell if this assay could be suitable for clinical use as well.

The proteases 2A and 3C, included in the panel, gave similar results to our previous study focusing on enterovirus-IgM negative and IgM positive adults [19]. In that study we found the antibodies against viral proteases as potent markers for an acute infection in adults. Here, we found that children often had high 3C reactivity, but low VP1 responses, indicating that either, protease specific antibodies would not serve as marker for acute infections in children or that the VP1 responses in children are more serotype specific than we expected. If the antibody responses to VP1s in children are indeed more serotype than species specific, then it could explain why we see high responses to the proteases which’ sequences are more conserved between enteroviruses than the VP1s’ [19,24]. This is plausible as rhinovirus infections are the most common virus associated with wheezing in children aged between one and two years according to a recent study [31]. Studying this phenomenon in high detail would require samples from children confirmed to be acutely infected.

The multiplex assay is very flexible and fast to run. The U-PLEX platform is typically used as a sandwich assay [32] and mostly for the detection and quantification of biomarkers, such as interleukins [33]. However, it is possible to use any biotinylated molecules for coating as we did in this study. The run time for two 96 well plates for this assay including sample preparation, coating and incubations was roughly 5 h for one person. Therefore, a multiplexed approach for studying large serum collections would greatly increase efficiency of cohort studies. The platform we chose is flexible for prototyping assays as we can easily try different combinations of antigens. This simple change could make the panel more relevant to Asian countries, where hand, foot and mouth disease caused primarily by EV-A71 and CVA16 is prevalent, while the rest of the panel stayed the same. The authors are unaware of any other immunology based multiplex assay for enteroviruses that has as broad coverage as the one we describe in the current study. This kind of multiplexed assay could solve many of the problems large scale studies such as DIPP [28] are facing. Because the assay is sensitive and requires small sample volumes, less hands-on work is required in the laboratory and a smaller number of plates needs to be run as opposed to regular ELISA. This also has the added benefit of reducing the effects of inter-plate variation.

Studying the cross-reactivity of enterovirus antibody responses using this multiplex assay is simple. Information on cross-reactivity could be important for studying prototype vaccines’ responses, or if aiming to produce more accurate assays by reducing cross-reactivity. In recent years the interest in vaccinating against enteroviruses that cause chronic diseases have gained more momentum and prototype vaccines are being developed [34,35]. One major source of cross-reactivity between enterovirus antibodies is the VP1 *N*-terminal enterovirus group-specific immunodominant epitope; however, it is not a neutralizing one [18]. A similar epitope has been found in rhinoviruses, which is highly conserved between them [36]. The presence of this epitope has been hypothesized to be one reason why vaccines for the common cold caused by the more than 150 rhinovirus serotypes has not been developed so far. Niespodziana and colleagues [36] discussed the possibility that as the antibody response towards this non-neutralizing epitope gets stronger with every new rhinovirus infection, diverting resources from making neutralizing antibodies similarly to the classical example first reported in influenza viruses and aptly termed the original antigenic sin [37,38].

There are a few possible strategies to make the assay less cross-reactive. One would involve removing the enterovirus and rhinovirus specific epitopes from VP1 antigens and another one could involve pre-incubating serum samples with said epitopes to reduce binding to the plated antigens. Using this panel of antigens for studying the IgM fraction of sera could also make the test more specific and relevant for diagnosing acute infections, as IgM responses have been shown to be less cross-reactive than IgG-response [39]. Studying only the IgM antibodies would however require a serum fractionation step prior to applying the samples to the assay if it is to stay on this platform. If we achieved better specificity, it could be then possible to distinguish between serotypes as well as species by looking at immune profile “fingerprint” patterns in antigen reactivity, revealing the recent infection history.

## 5. Conclusions

We have developed a novel multiplex immunoassay covering all the enterovirus species. Using human samples from confirmed enterovirus-infected and healthy individuals, we have shown that the antibody responses towards different enteroviruses and rhinoviruses are more species specific in children than in adults. We have also shown that the developed multiplex assay is versatile and after further validation it has potential to find use in both patient diagnostics as well as for studying pathogen-disease connections associated with enteroviruses and rhinoviruses in large cohort studies.

## Figures and Tables

**Table 1:** Summary of sample groups.

Sample Group	*n*
Adult IgM pos ^1^	20
Adult IgM neg ^1^	22
Adult biobank	20
Children ^2^	88

^1^ IgM tested with enteroviruses (EV)-A and EV-B VP1 proteins [19]; ^2^ Seroneutralization assays performed for various EV-A and EV-B viruses [15].

**Table 2:** Results of Wilcoxon rank-sum test between IgM positive and negative adults.

Antigen	*p*	*p* Adj.^1^
CVA4	0.113	N.S.
CVB1	0.167	N.S.
PV1	0.082	N.S.
EV-D68	**0.048**	0.477
RV-A89	0.252	N.S.
RV-B14	0.167	N.S.
RV-C3	0.116	N.S.
2A	**0.003**	**0.032**
3C	**0.030**	0.296
BSA	0.470	N.S.

^1^ Bonferroni correction. Statistically significant results bolded.

## Appendix A

**Table A1:** Details on VP1 antigens.

Species	Serotype	Genbank	MW (kDa)	PI	Lysines
EV-A	CVA4	AY421762.1	61.5	7.0	30
EV-B	CVB1	AB646478.1^1^	58.9	8.65	33
EV-C	PV1	V01150.1	60.9	7.3	37
EV-D	EV-D68	EF107098.1	61.7	7.78	38
RV-A	RV-A89	M16248.1	61.7	7.68	38
RV-B	RV-B14	AY355195.1	59.9	6.52	40
RV-C	RV-C3	EF186077.2	58.2	6.23	36

Closest sequence on Genbank, the exact sequence is same as in [14].

**Table A2:** Complete amino acid sequences of the recombinant VP1 antigens. VP1 parts unique to each antigen are typed in **bold.**

Antigen	Sequence
CVA4 VP1	MSPILGYWKIKGLVQPTRLLLEYLEEKYEEHLYERDEGDKWRNKKFELGLEFPNLPYYIDGDVKLTQSMAIIRYIADKHNMLGGCPKERAEISMLEGAVLDIRYGVSRIAYSKDFETLKVDFLSKLPEMLKMFEDRLCHKTYLNGDHVTHPDFMLYDALDVVLYMDPMCLDAFPKLVCFKKRIEAIPQIDKYLKSSKYIAWPLQGWQATFGGGDHPPKSDLVPRGS**GDAIADAIQNTVTSTIQRVTTNTVGQDATAANTAPSSHSLNTGLVPALQAAETGASSTATDGNLIETRCVVNSNGTRETHIEHFFSRSGLVGVMEVDDTGTSGKGFSNWDIDIMAFVQLRRKLEAFTYMRFDAEFTFVTNLENGLTNNSVIQYMYVPPGAPKPDARESFQWQTATNPSVFQKMDSPPPQVSVPFMSPASAYQWFYDGYPTFGPHSETSNLSYGQCPNNMLGTFSARVVSKQITNQKFQIRIYLRLKRVRAWIPRPLRSQPYIYRNYPTYGTTIQYLAKDRRKITETDYNAEQR** **TH**PGHHHHHHPG
CVB1 VP1	MSPILGYWKIKGLVQPTRLLLEYLEEKYEEHLYERDEGDKWRNKKFELGLEFPNLPYYIDGDVKLTQSMAIIRYIADKHNMLGGCPKERAEISMLEGAVLDIRYGVSRIAYSKDFETLKVDFLSKLPEMLKMFEDRLCHKTYLNGDHVTHPDFMLYDALDVVLYMDPMCLDAFPKLVCFKKRIEAIPQIDKYLKSSKYIAWPLQGWQATFGGGDHPPKSDLVPRGS**GPVEESVERAMVRVADTVSSKPTNSESIPALTAAETGHTSQVVPSDTMQTRHVKNYHSRSESSIENFLCRSACVYYATYTNNSKKGYAEWVINTRQVAQLRRKLELFTYLRFDLELTFVITSAQQPSTATSVDAPVQTHQIMYVPPGGPVPTKVTDYAWQTSTNPSVFWTEGNAPPRMSIPFISIGNAYSCFYDGWTQFSRNGVYGINTLNNMGTLYMRHVNEAGQGPIKSTVRIYFKPKHVKAWVPRPP** **RLCQYEKQKNVNFNPTGVTTTRSNITTTGAF**PGHHHHHHPG
PV1 VP1	MSPILGYWKIKGLVQPTRLLLEYLEEKYEEHLYERDEGDKWRNKKFELGLEFPNLPYYIDGDVKLTQSMAIIRYIADKHNMLGGCPKERAEISMLEGAVLDIRYGVSRIAYSKDFETLKVDFLSKLPEMLKMFEDRLCHKTYLNGDHVTHPDFMLYDALDVVLYMDPMCLDAFPKLVCFKKRIEAIPQIDKYLKSSKYIAWPLQGWQATFGGGDHPPKSDLVPRGS**GLGQMLESMIDNTVRETVGAATSRDALPNTEASGPAHSKEIPALTAVETGATNPLVPSDTVQTRHVVQHRSRSESSIESFFARGACVAIITVDNSASTKNKDKLFTVWKITYKDTVQLRRKLEFFTYSRFDMEFTFVVTANFTETNNGHALNQVYQIMYVPPGAPVPEKWDDYTWQTSSNPSIFYTYGTAPARISVPYVGISNAYSHFYDGFSKVPLKDQSAALGDSLYGAASLNDFGILAVRVVNDHNPTKVTSKIRVYLKPKHIRVWCPRPPRAVAYYGPGVDYKDGTLTPLSTKDLTTY**P GHHHHHHPG
EV-D68 VP1	MSPILGYWKIKGLVQPTRLLLEYLEEKYEEHLYERDEGDKWRNKKFELGLEFPNLPYYIDGDVKLTQSMAIIRYIADKHNMLGGCPKERAEISMLEGAVLDIRYGVSRIAYSKDFETLKVDFLSKLPEMLKMFEDRLCHKTYLNGDHVTHPDFMLYDALDVVLYMDPMCLDAFPKLVCFKKRIEAIPQIDKYLKSSKYIAWPLQGWQATFGGGDHPPKSDLVPRGS**LDHLDAAEAAYQIESIIKTATDTVKSEISAELGVVPSLNAVETGASSNTEPEEAIQTRTVINQHGVSETLVENFLSRAALVSKRSFEYKNHTSSKARTDKNFFKWTINTKSFVQLRRKLELFTYLRFDAEITILTTVAVNGSSNSTYMGLPDLTLQAMFVPTGALTPEKQDSFHWQSGSNASVFFKISDPPARMTIPFMCINSAYSVFYDGFAGFEKSGLYGINPADTIGNLCVRIVNEHQPIGFTVTVRVYMKPKHIKAWAPRPPRTLPYMSIANANYRGKDRAPNALNAIIGNRESVKTMPHNI** **VTT**PGHHHHHHPG
RV-A89 VP1	MSPILGYWKIKGLVQPTRLLLEYLEEKYEEHLYERDEGDKWRNKKFELGLEFPNLPYYIDGDVKLTQSMAIIRYIADKHNMLGGCPKERAEISMLEGAVLDIRYGVSRIAYSKDFETLKVDFLSKLPEMLKMFEDRLCHKTYLNGDHVTHPDFMLYDALDVVLYMDPMCLDAFPKLVCFKKRIEAIPQIDKYLKSSKYIAWPLQGWQATFGGGDHPPKSDLVPRGS**LDHLDAAEAAYQIESIIKTATDTVKSEISAELGVVPSLNAVETGASSNTEPEEAIQTRTVINQHGVSETLVENFLSRAALVSKRSFEYKNHTSSKARTDKNFFKWTINTKSFVQLRRKLELFTYLRFDAEITILTTVAVNGSSNSTYMGLPDLTLQAMFVPTGALTPEKQDSFHWQSGSNASVFFKISDPPARMTIPFMCINSAYSVFYDGFAGFEKSGLYGINPADTIGNLCVRIVNEHQPIGFTVTVRVYMKPKHIKAWAPRPPRTLPYMSIANANYRGKDRAPNALNAIIGNRESVKTMPHNI** **VTT**PGHHHHHHPG
RV-B14 VP1	MSPILGYWKIKGLVQPTRLLLEYLEEKYEEHLYERDEGDKWRNKKFELGLEFPNLPYYIDGDVKLTQSMAIIRYIADKHNMLGGCPKERAEISMLEGAVLDIRYGVSRIAYSKDFETLKVDFLSKLPEMLKMFEDRLCHKTYLNGDHVTHPDFMLYDALDVVLYMDPMCLDAFPKLVCFKKRIEAIPQIDKYLKSSKYIAWPLQGWQATFGGGDHPPKSDLVPRGS**GLGDELEEVIVEKTKQTVASISSGPKHTQKVPILTANETGATMPVLPSDSIETRTTYMHFNGSETDVECFLGRAACVHVTEIQNKDATGIDNHREAKLFNDWKINLSSLVQLRKKLELFTYVRFDSEYTILATASQPDSANYSSNLVVQAMYVPPGAPNPKEWDDYTWQSASNPSVFFKVGDTSRFSVPYVGLASAYNCFYDGYSHDDAETQYGITVLNHMGSMAFRIVNEHDEHKTLVKIRVYHRAKHV** **EAWIPRAPRALPYTSIGRTNYPKNTEPVIKKRKGDIKSY**PGHHHHHHPG
RV-C3 VP1	MSPILGYWKIKGLVQPTRLLLEYLEEKYEEHLYERDEGDKWRNKKFELGLEFPNLPYYIDGDVKLTQSMAIIRYIADKHNMLGGCPKERAEISMLEGAVLDIRYGVSRIAYSKDFETLKVDFLSKLPEMLKMFEDRLCHKTYLNGDHVTHPDFMLYDALDVVLYMDPMCLDAFPKLVCFKKRIEAIPQIDKYLKSSKYIAWPLQGWQATFGGGDHPPKSDLVPRGS**NPVEEFVEHTLKEVLVVPDTQASGPVHTTKPQALGAVEIGATADVGPETLIETRYVMNDNTNAEAAVENFLGRSALWANLRLDQGFRKWEINFQEHAQVRKKFEMFTYVRFDLEITIVTNNKGLMQIMFVPPGITPPGGKDGREWDTASNPSVFFQPNSGFPRFTIPFTGLGSAYYMFYDGYDGTDDANINYGISLTNDMGTLCFRALDGTGASDIKVFGKPKHITAWIPRPPRATQYLHKFSTNYNKPK** **TSGSTELEPKHFFKYRQDITSITNL**PGHHHHHHPG
