# Correction: Saarinen, N.V.V., et al. Multiplexed High-Throughput Serological Assay for Human Enteroviruses. *Microorganismis* 2020, *8*, 963

**DOI:** 10.3390/microorganisms8091426

**Published:** 2020-09-17

**Authors:** Niila V. V. Saarinen, Jussi Lehtonen, Riitta Veijola, Johanna Lempainen, Mikael Knip, Heikki Hyöty, Olli H. Laitinen, Vesa P. Hytönen

**Affiliations:** 1 Faculty of Medicine and Health Technology, Tampere University, 33520 Tampere, Finland; niila.saarinen@tuni.fi (N.V.V.S.); jussi.lehtonen@tuni.fi (J.L.); heikki.hyoty@tuni.fi (H.H.); olli.laitinen@tuni.fi (O.H.L.); 2 Department of Paediatrics, University of Oulu, 90570 Oulu, Finland; riitta.veijola@oulu.fi; 3 Department of Paediatrics, University of Turku, 20520 Turku, Finland; johanna.lempainen@utu.fi; 4 Pediatric Research Center, Children’s Hospital, University of Helsinki and Helsinki University Hospital, and Research Program for Clinical and Molecular Metabolism, Faculty of Medicine, University of Helsinki, 00029 Helsinki, Finland; mikael.knip@helsinki.fi; 5 Fimlab Laboratories, 33520 Tampere, Finland

The authors wish to make the following correction to this paper [1]: The protein sequence corresponding to RV-A89 VP1 was erroneous in Table A2.

**Table A2:** Complete amino acid sequences of the recombinant VP1 antigens. VP1 parts unique to each antigen are typed in **bold.**

Antigen	Sequence
CVA4 VP1	MSPILGYWKIKGLVQPTRLLLEYLEEKYEEHLYERDEGDKWRNKKFELGLEFPNLPYYIDGDVKLTQSMAIIRYIADKHNMLGGCPKERAEISMLEGAVLDIRYGVSRIAYSKDFETLKVDFLSKLPEMLKMFEDRLCHKTYLNGDHVTHPDFMLYDALDVVLYMDPMCLDAFPKLVCFKKRIEAIPQIDKYLKSSKYIAWPLQGWQATFGGGDHPPKSDLVPRGS**GDAIADAIQNTVTSTIQRVTTNTVGQDATAANTAPSSHSLNTGLVPALQAAETGASSTATDGNLIETRCVVNSNGTRETHIEHFFSRSGLVGVMEVDDTGTSGKGFSNWDIDIMAFVQLRRKLEAFTYMRFDAEFTFVTNLENGLTNNSVIQYMYVPPGAPKPDARESFQWQTATNPSVFQKMDSPPPQVSVPFMSPASAYQWFYDGYPTFGPHSETSNLSYGQCPNNMLGTFSARVVSKQITNQKFQIRIYLRLKRVRAWIPRPLRSQPYIYRNYPTYGTTIQYLAKDRRKITETDYNAEQR** **TH**PGHHHHHHPG
CVB1 VP1	MSPILGYWKIKGLVQPTRLLLEYLEEKYEEHLYERDEGDKWRNKKFELGLEFPNLPYYIDGDVKLTQSMAIIRYIADKHNMLGGCPKERAEISMLEGAVLDIRYGVSRIAYSKDFETLKVDFLSKLPEMLKMFEDRLCHKTYLNGDHVTHPDFMLYDALDVVLYMDPMCLDAFPKLVCFKKRIEAIPQIDKYLKSSKYIAWPLQGWQATFGGGDHPPKSDLVPRGS**GPVEESVERAMVRVADTVSSKPTNSESIPALTAAETGHTSQVVPSDTMQTRHVKNYHSRSESSIENFLCRSACVYYATYTNNSKKGYAEWVINTRQVAQLRRKLELFTYLRFDLELTFVITSAQQPSTATSVDAPVQTHQIMYVPPGGPVPTKVTDYAWQTSTNPSVFWTEGNAPPRMSIPFISIGNAYSCFYDGWTQFSRNGVYGINTLNNMGTLYMRHVNEAGQGPIKSTVRIYFKPKHVKAWVPRPP** **RLCQYEKQKNVNFNPTGVTTTRSNITTTGAF**PGHHHHHHPG
PV1 VP1	MSPILGYWKIKGLVQPTRLLLEYLEEKYEEHLYERDEGDKWRNKKFELGLEFPNLPYYIDGDVKLTQSMAIIRYIADKHNMLGGCPKERAEISMLEGAVLDIRYGVSRIAYSKDFETLKVDFLSKLPEMLKMFEDRLCHKTYLNGDHVTHPDFMLYDALDVVLYMDPMCLDAFPKLVCFKKRIEAIPQIDKYLKSSKYIAWPLQGWQATFGGGDHPPKSDLVPRGS**GLGQMLESMIDNTVRETVGAATSRDALPNTEASGPAHSKEIPALTAVETGATNPLVPSDTVQTRHVVQHRSRSESSIESFFARGACVAIITVDNSASTKNKDKLFTVWKITYKDTVQLRRKLEFFTYSRFDMEFTFVVTANFTETNNGHALNQVYQIMYVPPGAPVPEKWDDYTWQTSSNPSIFYTYGTAPARISVPYVGISNAYSHFYDGFSKVPLKDQSAALGDSLYGAASLNDFGILAVRVVNDHNPTKVTSKIRVYLKPKHIRVWCPRPPRAVAYYGPGVDYKDGTLTPLSTKDLTTY**P GHHHHHHPG
EV-D68 VP1	MSPILGYWKIKGLVQPTRLLLEYLEEKYEEHLYERDEGDKWRNKKFELGLEFPNLPYYIDGDVKLTQSMAIIRYIADKHNMLGGCPKERAEISMLEGAVLDIRYGVSRIAYSKDFETLKVDFLSKLPEMLKMFEDRLCHKTYLNGDHVTHPDFMLYDALDVVLYMDPMCLDAFPKLVCFKKRIEAIPQIDKYLKSSKYIAWPLQGWQATFGGGDHPPKSDLVPRGS**LDHLDAAEAAYQIESIIKTATDTVKSEISAELGVVPSLNAVETGASSNTEPEEAIQTRTVINQHGVSETLVENFLSRAALVSKRSFEYKNHTSSKARTDKNFFKWTINTKSFVQLRRKLELFTYLRFDAEITILTTVAVNGSSNSTYMGLPDLTLQAMFVPTGALTPEKQDSFHWQSGSNASVFFKISDPPARMTIPFMCINSAYSVFYDGFAGFEKSGLYGINPADTIGNLCVRIVNEHQPIGFTVTVRVYMKPKHIKAWAPRPPRTLPYMSIANANYRGKDRAPNALNAIIGNRESVKTMPHNI** **VTT**PGHHHHHHPG
RV-A89 VP1	MSPILGYWKIKGLVQPTRLLLEYLEEKYEEHLYERDEGDKWRNKKFELGLEFPNLPYYIDGDVKLTQSMAIIRYIADKHNMLGGCPKERAEISMLEGAVLDIRYGVSRIAYSKDFETLKVDFLSKLPEMLKMFEDRLCHKTYLNGDHVTHPDFMLYDALDVVLYMDPMCLDAFPKLVCFKKRIEAIPQIDKYLKSSKYIAWPLQGWQATFGGGDHPPKSDLVPRGS **LDHLDAAEAAYQIESIIKTATDTVKSEISAELGVVPSLNAVETGASSNTEPEEAIQTRTVINQHGVSETLVENFLSRAALVSKRSFEYKNHTSSKARTDKNFFKWTINTKSFVQLRRKLELFTYLRFDAEITILTTVAVNGSSNSTYMGLPDLTLQAMFVPTGALTPEKQDSFHWQSGSNASVFFKISDPPARMTIPFMCINSAYSVFYDGFAGFEKSGLYGINPADTIGNLCVRIVNEHQPIGFTVTVRVYMKPKHIKAWAPRPPRTLPYMSIANANYRGKDRAPNALNAIIGNRESVKTMPHNIVTT** PGHHHHHHPG
RV-B14 VP1	MSPILGYWKIKGLVQPTRLLLEYLEEKYEEHLYERDEGDKWRNKKFELGLEFPNLPYYIDGDVKLTQSMAIIRYIADKHNMLGGCPKERAEISMLEGAVLDIRYGVSRIAYSKDFETLKVDFLSKLPEMLKMFEDRLCHKTYLNGDHVTHPDFMLYDALDVVLYMDPMCLDAFPKLVCFKKRIEAIPQIDKYLKSSKYIAWPLQGWQATFGGGDHPPKSDLVPRGS**GLGDELEEVIVEKTKQTVASISSGPKHTQKVPILTANETGATMPVLPSDSIETRTTYMHFNGSETDVECFLGRAACVHVTEIQNKDATGIDNHREAKLFNDWKINLSSLVQLRKKLELFTYVRFDSEYTILATASQPDSANYSSNLVVQAMYVPPGAPNPKEWDDYTWQSASNPSVFFKVGDTSRFSVPYVGLASAYNCFYDGYSHDDAETQYGITVLNHMGSMAFRIVNEHDEHKTLVKIRVYHRAKHV** **EAWIPRAPRALPYTSIGRTNYPKNTEPVIKKRKGDIKSY**PGHHHHHHPG
RV-C3 VP1	MSPILGYWKIKGLVQPTRLLLEYLEEKYEEHLYERDEGDKWRNKKFELGLEFPNLPYYIDGDVKLTQSMAIIRYIADKHNMLGGCPKERAEISMLEGAVLDIRYGVSRIAYSKDFETLKVDFLSKLPEMLKMFEDRLCHKTYLNGDHVTHPDFMLYDALDVVLYMDPMCLDAFPKLVCFKKRIEAIPQIDKYLKSSKYIAWPLQGWQATFGGGDHPPKSDLVPRGS**NPVEEFVEHTLKEVLVVPDTQASGPVHTTKPQALGAVEIGATADVGPETLIETRYVMNDNTNAEAAVENFLGRSALWANLRLDQGFRKWEINFQEHAQVRKKFEMFTYVRFDLEITIVTNNKGLMQIMFVPPGITPPGGKDGREWDTASNPSVFFQPNSGFPRFTIPFTGLGSAYYMFYDGYDGTDDANINYGISLTNDMGTLCFRALDGTGASDIKVFGKPKHITAWIPRPPRATQYLHKFSTNYNKPK** **TSGSTELEPKHFFKYRQDITSITNL**PGHHHHHHPG

To the correct version, as follows:

**Table A2:** Complete amino acid sequences of the recombinant VP1 antigens. VP1 parts unique to each antigen are typed in **bold.**

Antigen	Sequence
CVA4 VP1	MSPILGYWKIKGLVQPTRLLLEYLEEKYEEHLYERDEGDKWRNKKFELGLEFPNLPYYIDGDVKLTQSMAIIRYIADKHNMLGGCPKERAEISMLEGAVLDIRYGVSRIAYSKDFETLKVDFLSKLPEMLKMFEDRLCHKTYLNGDHVTHPDFMLYDALDVVLYMDPMCLDAFPKLVCFKKRIEAIPQIDKYLKSSKYIAWPLQGWQATFGGGDHPPKSDLVPRGS**GDAIADAIQNTVTSTIQRVTTNTVGQDATAANTAPSSHSLNTGLVPALQAAETGASSTATDGNLIETRCVVNSNGTRETHIEHFFSRSGLVGVMEVDDTGTSGKGFSNWDIDIMAFVQLRRKLEAFTYMRFDAEFTFVTNLENGLTNNSVIQYMYVPPGAPKPDARESFQWQTATNPSVFQKMDSPPPQVSVPFMSPASAYQWFYDGYPTFGPHSETSNLSYGQCPNNMLGTFSARVVSKQITNQKFQIRIYLRLKRVRAWIPRPLRSQPYIYRNYPTYGTTIQYLAKDRRKITETDYNAEQR** **TH**PGHHHHHHPG
CVB1 VP1	MSPILGYWKIKGLVQPTRLLLEYLEEKYEEHLYERDEGDKWRNKKFELGLEFPNLPYYIDGDVKLTQSMAIIRYIADKHNMLGGCPKERAEISMLEGAVLDIRYGVSRIAYSKDFETLKVDFLSKLPEMLKMFEDRLCHKTYLNGDHVTHPDFMLYDALDVVLYMDPMCLDAFPKLVCFKKRIEAIPQIDKYLKSSKYIAWPLQGWQATFGGGDHPPKSDLVPRGS**GPVEESVERAMVRVADTVSSKPTNSESIPALTAAETGHTSQVVPSDTMQTRHVKNYHSRSESSIENFLCRSACVYYATYTNNSKKGYAEWVINTRQVAQLRRKLELFTYLRFDLELTFVITSAQQPSTATSVDAPVQTHQIMYVPPGGPVPTKVTDYAWQTSTNPSVFWTEGNAPPRMSIPFISIGNAYSCFYDGWTQFSRNGVYGINTLNNMGTLYMRHVNEAGQGPIKSTVRIYFKPKHVKAWVPRPP** **RLCQYEKQKNVNFNPTGVTTTRSNITTTGAF**PGHHHHHHPG
PV1 VP1	MSPILGYWKIKGLVQPTRLLLEYLEEKYEEHLYERDEGDKWRNKKFELGLEFPNLPYYIDGDVKLTQSMAIIRYIADKHNMLGGCPKERAEISMLEGAVLDIRYGVSRIAYSKDFETLKVDFLSKLPEMLKMFEDRLCHKTYLNGDHVTHPDFMLYDALDVVLYMDPMCLDAFPKLVCFKKRIEAIPQIDKYLKSSKYIAWPLQGWQATFGGGDHPPKSDLVPRGS**GLGQMLESMIDNTVRETVGAATSRDALPNTEASGPAHSKEIPALTAVETGATNPLVPSDTVQTRHVVQHRSRSESSIESFFARGACVAIITVDNSASTKNKDKLFTVWKITYKDTVQLRRKLEFFTYSRFDMEFTFVVTANFTETNNGHALNQVYQIMYVPPGAPVPEKWDDYTWQTSSNPSIFYTYGTAPARISVPYVGISNAYSHFYDGFSKVPLKDQSAALGDSLYGAASLNDFGILAVRVVNDHNPTKVTSKIRVYLKPKHIRVWCPRPPRAVAYYGPGVDYKDGTLTPLSTKDLTTY**P GHHHHHHPG
EV-D68 VP1	MSPILGYWKIKGLVQPTRLLLEYLEEKYEEHLYERDEGDKWRNKKFELGLEFPNLPYYIDGDVKLTQSMAIIRYIADKHNMLGGCPKERAEISMLEGAVLDIRYGVSRIAYSKDFETLKVDFLSKLPEMLKMFEDRLCHKTYLNGDHVTHPDFMLYDALDVVLYMDPMCLDAFPKLVCFKKRIEAIPQIDKYLKSSKYIAWPLQGWQATFGGGDHPPKSDLVPRGS**LDHLDAAEAAYQIESIIKTATDTVKSEISAELGVVPSLNAVETGASSNTEPEEAIQTRTVINQHGVSETLVENFLSRAALVSKRSFEYKNHTSSKARTDKNFFKWTINTKSFVQLRRKLELFTYLRFDAEITILTTVAVNGSSNSTYMGLPDLTLQAMFVPTGALTPEKQDSFHWQSGSNASVFFKISDPPARMTIPFMCINSAYSVFYDGFAGFEKSGLYGINPADTIGNLCVRIVNEHQPIGFTVTVRVYMKPKHIKAWAPRPPRTLPYMSIANANYRGKDRAPNALNAIIGNRESVKTMPHNI** **VTT**PGHHHHHHPG
RV-A89 VP1	MSPILGYWKIKGLVQPTRLLLEYLEEKYEEHLYERDEGDKWRNKKFELGLEFPNLPYYIDGDVKLTQSMAIIRYIADKHNMLGGCPKERAEISMLEGAVLDIRYGVSRIAYSKDFETLKVDFLSKLPEMLKMFEDRLCHKTYLNGDHVTHPDFMLYDALDVVLYMDPMCLDAFPKLVCFKKRIEAIPQIDKYLKSSKYIAWPLQGWQATFGGGDHPPKSDLVPRGS**NPVENYIDSVLNEVLVVPNIQPSTSVSSHAAPALDAAETGHTSSVQPEDMIETRYVITDQTRDETSIESFLGRSGCIAMIEFNTSSDKTEHDKIGKGFKTWKVSLQEMAQIRRKYELFTYTRFDSEITIVTAAAAQGNDSGHIVLQFMYVPPGAPVPEKRDDYTWQSGTNASVFWQEGQPYPRFTIPFMSIASAYYMFYDGYDGDSAASKYGSVVTNDMGTICVRIVTSNQKHDSNIVCRIYHKAKHIKAWCPRPPRAVAYQHTHSTNYIPSNGEATTQIKTRPDVFTVTNVGPSSMF**PGHHHHHHPG
RV-B14 VP1	MSPILGYWKIKGLVQPTRLLLEYLEEKYEEHLYERDEGDKWRNKKFELGLEFPNLPYYIDGDVKLTQSMAIIRYIADKHNMLGGCPKERAEISMLEGAVLDIRYGVSRIAYSKDFETLKVDFLSKLPEMLKMFEDRLCHKTYLNGDHVTHPDFMLYDALDVVLYMDPMCLDAFPKLVCFKKRIEAIPQIDKYLKSSKYIAWPLQGWQATFGGGDHPPKSDLVPRGS**GLGDELEEVIVEKTKQTVASISSGPKHTQKVPILTANETGATMPVLPSDSIETRTTYMHFNGSETDVECFLGRAACVHVTEIQNKDATGIDNHREAKLFNDWKINLSSLVQLRKKLELFTYVRFDSEYTILATASQPDSANYSSNLVVQAMYVPPGAPNPKEWDDYTWQSASNPSVFFKVGDTSRFSVPYVGLASAYNCFYDGYSHDDAETQYGITVLNHMGSMAFRIVNEHDEHKTLVKIRVYHRAKHV** **EAWIPRAPRALPYTSIGRTNYPKNTEPVIKKRKGDIKSY**PGHHHHHHPG
RV-C3 VP1	MSPILGYWKIKGLVQPTRLLLEYLEEKYEEHLYERDEGDKWRNKKFELGLEFPNLPYYIDGDVKLTQSMAIIRYIADKHNMLGGCPKERAEISMLEGAVLDIRYGVSRIAYSKDFETLKVDFLSKLPEMLKMFEDRLCHKTYLNGDHVTHPDFMLYDALDVVLYMDPMCLDAFPKLVCFKKRIEAIPQIDKYLKSSKYIAWPLQGWQATFGGGDHPPKSDLVPRGS**NPVEEFVEHTLKEVLVVPDTQASGPVHTTKPQALGAVEIGATADVGPETLIETRYVMNDNTNAEAAVENFLGRSALWANLRLDQGFRKWEINFQEHAQVRKKFEMFTYVRFDLEITIVTNNKGLMQIMFVPPGITPPGGKDGREWDTASNPSVFFQPNSGFPRFTIPFTGLGSAYYMFYDGYDGTDDANINYGISLTNDMGTLCFRALDGTGASDIKVFGKPKHITAWIPRPPRATQYLHKFSTNYNKPK** **TSGSTELEPKHFFKYRQDITSITNL**PGHHHHHHPG

The authors would like to apologize for any inconvenience caused to the readers by this change.
